# Flexible All-Carbon Nanoarchitecture Built from In Situ Formation of Nanoporous Graphene Within “Skeletal-Capillary” Carbon Nanotube Networks for Supercapacitors

**DOI:** 10.3390/nano14201683

**Published:** 2024-10-21

**Authors:** Tao Chen, Hongyan Li, Jiaziyi Wang, Xilai Jia

**Affiliations:** 1 State Grid Jilin Electric Power Research Institute, Changchun 130021, China; 2 School of Materials Science and Engineering, University of Science and Technology Beijing, Beijing 100083, China

**Keywords:** carbon materials, carbon nanotubes, graphene, energy storage

## Abstract

It is difficult for carbonaceous materials to combine a large specific surface area with flexibility. Here, a flexible all-carbon nanoarchitecture based on the in situ growth of nanoporous graphene within “skeletal-capillary” carbon nanotube (CNT) networks has been achieved by a chemical vapor deposition (CVD) process. Multi-path long-range conductivity is established, and the porous graphene provides a large specific surface area for charge storage. The flexibility of the films allows them to be directly used as binder-free electrodes for supercapacitors. Since the polymeric binders are saved, the supercapacitors exhibit a higher overall storage density.

## 1. Introduction

The supercapacitor is a very important electrochemical device. The activated carbon has achieved commercial success in supercapacitor devices [1]. They offer a number of desirable properties, including high power density and lifetime. However, current supercapacitors suffer from low energy density (typically 5–10 Wh kg^−1^). Accordingly, extensive efforts have been devoted to developing new carbon materials [2,3], such as CNTs and graphene with unique properties. In particular, one-dimensional (1D) CNTs are easily self-supported in networks and offer long-range electrical conductivity due to the high aspect ratios [4]; whereas, two-dimensional (2D) graphene is a promising material for boosting energy density as it is atomically thin [5]. The rational combination of these two is expected to create composite electrodes with better performance.

Due to good electrical conductivity and high porosity, which are important for ion diffusion and charge transfer, it is generally believed that graphene–CNT hybrids can bring on enhanced advantages over the respective constituents [6,7,8]. A common method to achieve this combination is direct mechanical mixing of the two. Despite the weak interfacial contacts, such a combination attains synergetic effects on the electrode performance of supercapacitor devices [9]. Alternatively, self-assembly is also available for preparing such composites based on hydrophilic/hydrophobic interactions, typically like layer-by-layer-assembled films [10]; however, the strength of such stacks is usually weak. For a better composite integration, combining the CVD of CNT and graphene together is another promising method [11]. Such composites have remarkable features of high conductivity and large surface areas, which are critical for supercapacitor applications. However, they always present as a powder that cannot be directly used as electrodes, which means extra components such as polymeric binders are required for a whole electrode.

Inspired by trees’ leaves that have high-efficiency water transport and conversion based on their hierarchical transport networks, herein, we have demonstrated the in situ growth of porous graphene within “skeletal-capillary” CNT networks to make flexible all-carbon nanoarchitecture with integrated structure. To construct “skeletal-capillary” CNT networks, two kinds of CNTs were used, that is, a millimeter-level CNT with a diameter of 30–50 nm and the other around 100-micrometer-long CNT with a diameter of 11 nm. The long-skeletal CNTs provide skeletons with long-range conductivity, while short-capillary CNTs entangle high-surface-area graphene with long-skeletal CNTs and offer conductive capillaries, which thereby form integrated all-carbon architecture. In this context, the obtained composites are freestanding and can be directly used as electrodes.

## 2. Experimental Methodology

### 2.1. Synthesis of the CNT/Graphene Nanocomposites

In this study, Mg(OH)_2_ was first synthesized using a surfactant-templated synthesis method. In detail, 1.0 g of MgO powder and 5.0 g of P123 (EO_20_PO_70_EO_20_ where EO and PO are ethylene oxide and propylene oxide, respectively) were dispersed into 1.0 L of deionized water and then boiled at 94 °C for 24 h, forming a uniform Mg(OH)_2_ suspension. The suspension was divided into 150, 310, and 530 mL, respectively. Then, skeletal CNTs and capillary CNTs with a weight ratio of 1:2 were dispersed into a homogeneous dispersion with the concentration at 0.05 and 0.1 mg mL^−1^, respectively, using a fluid shearing dispersion, into which an appropriate amount of Mg(OH)_2_ suspension was then added. The mixture dispersion was filtrated to form self-supported films, which were placed into a tube furnace. In a 100 mL min^−1^ of argon flow, the films were annealed at 550 °C for 1 h and then treated using a methane-CVD synthesis process at 900 °C for 10 min. After natural cooling, the produced films were immersed in 24% hydrochloric acid for 24 h to remove the residual MgO. The collected films were pressed by 1.0 Mpa and dried at 600 °C for 30 min, forming freestanding ternary CNT/graphene nanocomposite films.

### 2.2. Material Characterization

Scanning electron microscopy (SEM) experiments were conducted on a JEOL JSM-7401F instrument (Japan electronics Co., Ltd., Japan) at 3.0 kV. Transmission electron microscopy (TEM) experiments were conducted on a JEOL JEM-2010 instrument (Japan electronics Co., Ltd, Japan) operated at 120.0 kV. EDX analysis was performed using an EDAX apparatus. X-ray diffraction (XRD) was conducted on a Rigaku D/Max-rB diffractometer with Cu-K_a_ radiation (λ = 1.54 Å). Nitrogen sorption isotherms were measured using the Autosorb-IQ2-MP-C system (Quantachrome, Florida, USA). Before measurements, the sample was degassed at 300 °C until a manifold pressure of 2 mmHg was reached. The specific surface areas were calculated by the Brunauer–Emmett–Teller (BET) method using an adsorption branch in a relative pressure range from 0.01 to 0.1. The pore size distributions (*D*_p_) were derived from the adsorption branch of isotherms using the DFT method. Mechanical tests were conducted on INSTRON 5843 (Instron, Boston, USA) with a speed of 1.0 mm min^−1^ at room temperature. For volume conductivities under bending, one end of the composite film was tightly fixed by using a solid copper while the other end was mobile to reach different bending angles. Then, the electrical resistances of the composite film were measured by a 266 Clamp Meter (Kanghai instrument Co., Ltd., China) and used for calculation of the volume conductivities. 

### 2.3. Electrochemical Performance

The CNT/graphene nanocomposite films were cut into desired sheets with the same weight as working electrodes, which were assembled into 2032-type coin cells for symmetric supercapacitors. The electrolyte was an ionic liquid 1-ethyl-3-methyl imidazolium tetrafluoroborate (EMIMBF_4_). The device assemblies were conducted in an argon-filled glove box. The galvanostatic charge/discharge measurements were carried out by the LAND CT2000 capacitor tester (Wuhan land electronics Co., Ltd., China). The CV and EIS measurements were carried out on a CHI 660C electrochemical workstation (CH instruments, China).

## 3. Results and Discussion

**Figure 1:**
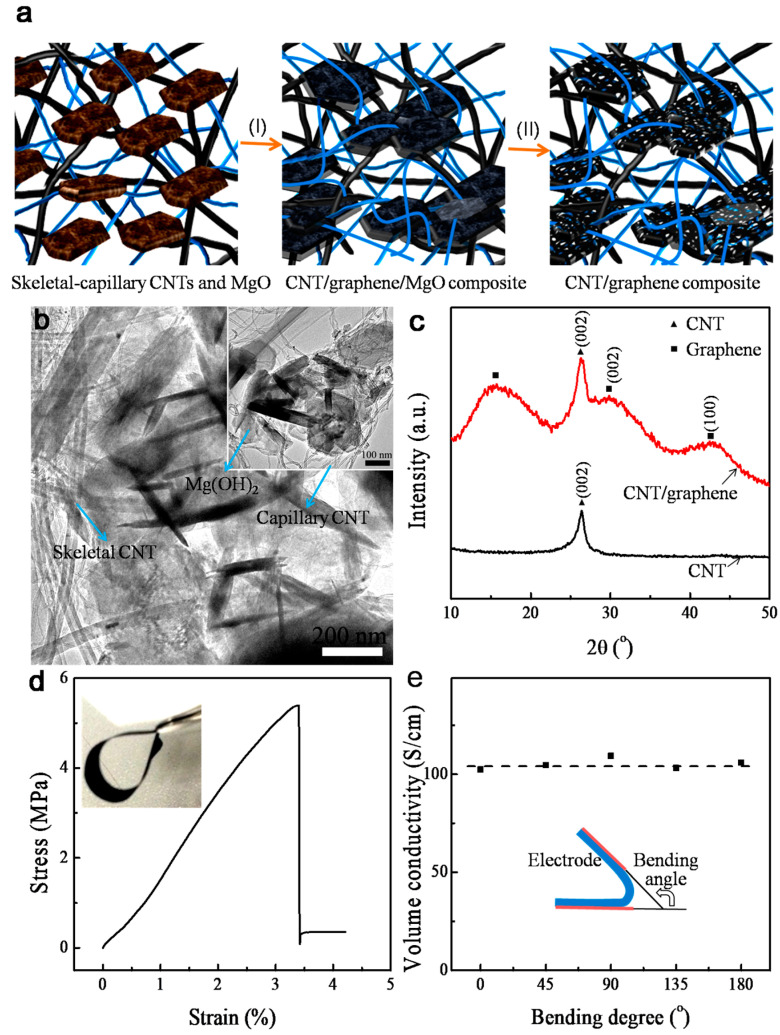
(**a**) Schematic fabrication of skeletal–capillary CNT/graphene composite. (**b**) SEM morphology of Mg(OH)_2_ in skeletal–capillary CNT networks. (**c**) XRD spectra of skeletal–capillary CNTs before and after in situ growth of porous graphene. (**d**) Stress–strain curve (inset showing a digital photograph of the flexible film) and (**e**) electrical conductivity of the electrode film.

Figure 1a outlines the approach: first, skeletal CNTs (Figure S1) and capillary CNTs (Figure S2) were dispersed in a homogeneous suspension, into which magnesium hydroxide (Mg(OH)_2_, Figure S3) dispersion was introduced as a graphene catalyst. The uniform mixture dispersion was filtrated to form self-supported films. Figure 1b shows that the Mg(OH)_2_ component can be well mixed into the skeletal–capillary CNT networks (weight ratios of skeletal CNTs:capillary CNTs:Mg(OH)_2_ = 1:2:54) after a high-speed shearing dispersion. The Mg(OH)_2_ powder was tightly entangled within the CNTs, particularly in capillary CNTs (inset in Figure 1b). After the methane-CVD at 900 °C for 10 min, XRD patterns (Figure 1c) conformed to the formation of porous graphene since the skeletal–capillary CNT/graphene composite displayed broad peaks at 2θ = 15.6°, 28.9°, and 42.9°, in contrast to the relatively sharp peak of CNTs (2θ = 26.4°). The broadness of the peak is ascribed to the disordered structure of as-grown porous graphene [12,13]. 

Figure 1d shows the flexibility of the freestanding composite films after removing the MgO component. The films displayed a tensile strength of 5.4 MPa at 3.4% strain, which was rarely reported for binder-free electrodes [14]. Such high mechanical strength indicates the tight integration between long-skeletal/short-capillary CNTs and as-grown graphene tissues. The intimate interfacial contacts are further confirmed through the electrical conductivity measurements under bending (Figure 1e), which offers a robust conductivity of around 102 S cm^−1^. To date, the integration of long and short CNTs is mainly achieved by simple mechanical mixing, such as the conductive agent in electrodes for lithium-ion batteries and partial supercapacitor devices. Here, the integrated combination of the two kinds of CNTs has been presented by the in situ growth of graphene. 

**Figure 2:**
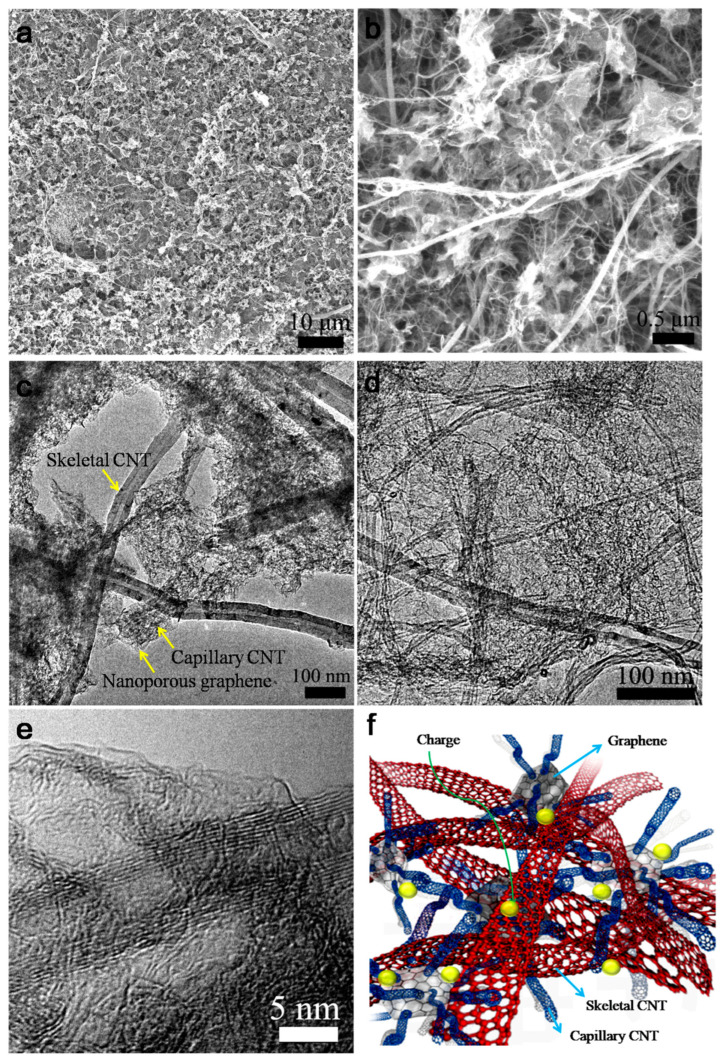
(**a**,**b**) SEM and (**c**–**e**) TEM images of the skeletal–capillary CNT/graphene composite. (**f**) Schematic of the charge transport in the skeletal–capillary CNT/graphene composite.

SEM characterization discloses that the skeletal–capillary CNT/graphene composites showed an integrated and uniform structure (Figure 2a), suggesting the good dispersion and integration of the building components. In the enlarged view, the as-grown graphene is highly entangled within the CNT networks (Figure 2b). The control experiments with only skeletal CNTs or capillary CNTs were also conducted. Figure S4a presents a loose morphology between CNTs and graphene when using only long-skeletal CNTs. The weak interactions of large-diameter skeletal CNTs were not so effective in preventing the aggregation of catalyst particles, thus offering loose integration. When using only capillary CNTs, partial aggregation of graphene can be observed (Figure S4b). Therefore, the rational combination of the two kinds of CNTs allows for better integration of the composite. Moreover, the composition of the skeletal–capillary CNT/graphene composite can be readily tuned by tuning the weight ratios of the Mg(OH)_2_ catalysts. When decreasing/increasing the amount of Mg(OH)_2_ component, the specific surface areas (SSA) of the as-fabricated composites are decreased/increased significantly as expected (Figure S5a and Table S1). Due to the increase in porous graphene, the intensity of the D peaks (assigned to the disordered carbon) in the Raman spectroscopy also increases for the nanocomposites (Figure S6). The SSA of the chosen skeletal–capillary CNT/graphene reaches 959.3 m^2^ g^−1^ while maintaining the 5.4 MPa strength. This supports that the nanoarchitecture combines a high specific surface area and mechanical flexibility. 

TEM characterization further confirms the interconnected morphology of the composite (Figure 2c,d), forming tight interfacial contacts. Different from simple mechanical mixing, the prepared nanocomposites achieve nanoscale dispersion and rational integration. Figure 2e reveals that the nanoporous graphene shows a crumpled feature that is different from flat or wrinkled sheets [15]. The crumpled pore structure is averaged at several nanometers with fewer than two graphene layers, as further confirmed by the pore-size distribution characterization (Figure S5b). Moreover, impurities such as iron are difficult to remove in the synthesis of the CNT/graphene composites, which may cause self-discharge or safety problems for energy storage devices; however, based on magnesium-oxide catalytic growth of graphene, no obvious metals were detected in the composites by energy-dispersive X-ray spectroscopy (EDX, Figure S7), which may offer safer devices. Overall, the flexible all-carbon composite is expected to provide effective electron and ion transport pathways for charge storage (Figure 2f).

**Figure 3:**
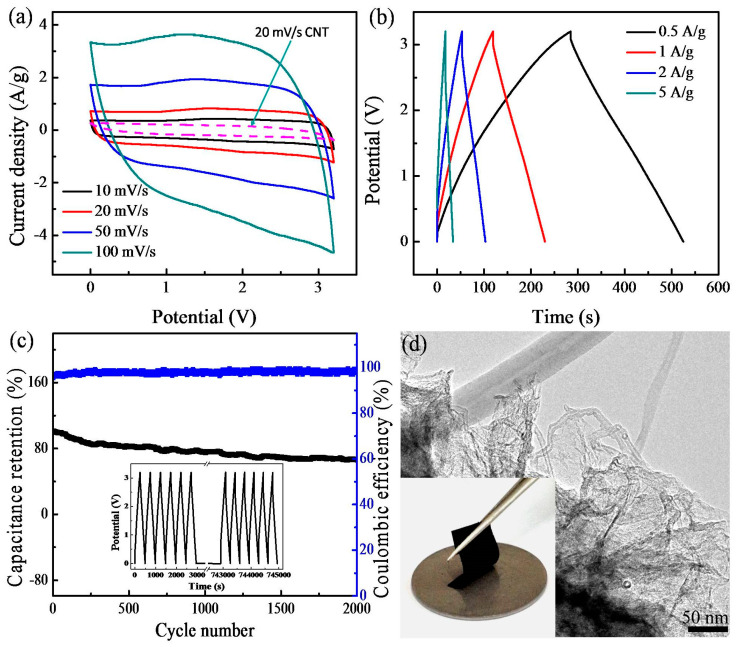
(**a**) CV plots. (**b**) Charge–discharge curves. (**c**) Cycling stability. (**d**) TEM image of the film electrode after 2000 cycles (inset: digital photograph).

As proof of the applications, the films were used as electrodes for symmetric supercapacitors using the EMIMBF_4_ as the electrolyte. Figure 3a illustrates the cyclic voltammetry (CV) curves of the supercapacitor behavior over scan rates of 10–100 mV s^−1^. The shapes of the CV curves of the samples indicate the double-layer capacitor nature [16]. Moreover, in the CV curves, a pair of broad peaks can be observed, which may be related to defect structures in the graphene and could contribute to pseudocapacitance [17,18]. Due to the high surface areas, the composite supercapacitor displayed much better capacity than the skeletal–capillary CNTs (e.g., 20 mV s^−1^). Figure 3b shows the galvanostatic charge–discharge profiles. The charging and discharging curves are basically symmetrical, suggesting the skeletal–capillary CNT/graphene supercapacitor achieves highly reversible energy storage. The full device could charge or discharge within ~112 s at a current of 1 A g^−1^, revealing fast kinetics. Figure 3c displays the cycling stability of the supercapacitor based on skeletal–capillary CNT/graphene electrodes. In spite of a capacitance decrease of ca. 15% of the initial capacitance that is attributed to the increased effective interfacial area between nanoporous graphene and electrolyte with the increase in reaction time, the supercapacitor shows relatively stable performance during the 2000 repeated cycles at 0.5 A g^−1^ (inset of Figure 3c: the initial and last six cycles’ charge–discharge curves). The Coulombic efficiency is maintained at ~99.8%. To further confirm the electrode structure integrity, after 2000 cycles, it was taken out of the supercapacitor and examined under the electron microscopy technique. As shown in Figure 3d, the electrode retains its macroscopic flexibility, and microscopically hierarchical porous structure, confirming its structural robustness.

Figure S8 further compares the Ragone plot of the designed symmetric supercapacitor. When discharged at 0.5 A g^−1^, an energy density of 53.1 Wh kg^−1^ was obtained with a power density of 800 W kg^−1^ based on the total mass of two electrodes. Even at a power density of 8000 W kg^−1^, the device still delivered an energy density of 35.6 Wh kg^−1^, an indication of high power and energy performance compared with the reported devices [19,20]. It needs to be pointed out that traditional electrodes contain a large number of polymeric binders that do not contribute to the capacity. The integrated CNT/graphene electrodes here can offer higher overall storage densities.

## 4. Conclusions

In conclusion, flexible all-carbon nanoarchitecture based on in situ growth of nanoporous graphene within “skeletal-capillary” CNT networks has been achieved. The nanoarchitecture provides multi-path long-range conductivity and a large specific surface area for charge storage. Moreover, the 5.4 MPa strength of the films allows them to be directly used as binder-free electrodes for supercapacitors. Due to the rational combination of CNTs and graphene features, the supercapacitor displays good energy density and power density.

## Data Availability

The original contributions presented in the study are included in the article/supplementary material, further inquiries can be directed to the corresponding author.

## Supplementary Materials

The following supporting information can be downloaded at: https://www.mdpi.com/article/10.3390/nano14201683/s1. References [21,22,23,24,25,26] are cited in the supplementary materials.
