# Supplementary material for: Flexible All-Carbon Nanoarchitecture Built from In Situ Formation of Nanoporous Graphene Within “Skeletal-Capillary” Carbon Nanotube Networks for Supercapacitors
Source: Nanomaterials (Basel). 2024 Oct 21;14(20):1683. doi: 10.3390/nano14201683 (PMC11510038; doi:10.3390/nano14201683)
Supplement: Supplementary file 1 [file nanomaterials-14-01683-s001.zip › nanomaterials-3160872-supplementary.pdf]

Electronic Supplementary Materials

Flexible All-Carbon Nanoarchitecture Built from In Situ Formation of Nanoporous Graphene Within "Skeletal-Capillary" Carbon Nanotube Networks for Supercapacitors

Tao Chen [1], Hongyan Li [1], Jiaziyi Wang [2], Xilai Jia [2,*]

[1] State Grid Jilin Electric Power Research Institute, Changchun 130021, China

[2] School of Materials Science and Engineering, University of Science and Technology Beijing, Beijing 100083, China

[*]Address correspondence to: jiaxl@ustb.edu.cn

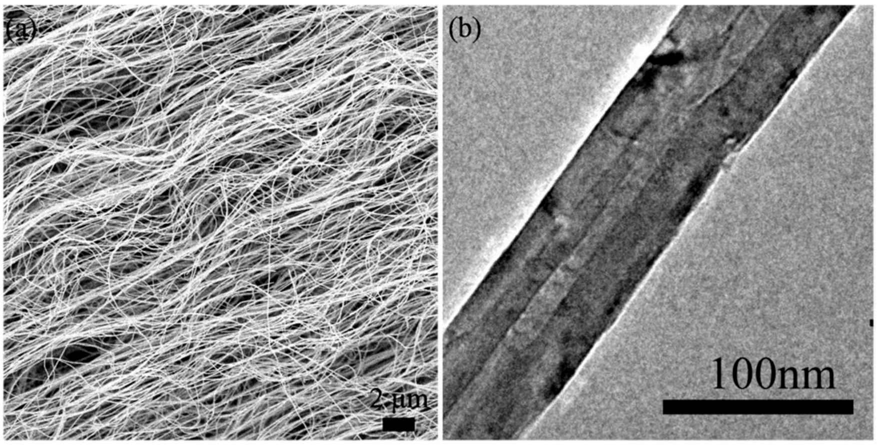

Figure S1 (a) SEM and (b) TEM images of long- skeletal CNTs. This kind of CNTs has the length up to half a millimeter level and the diameter at 30-50 nm. More details of such CNTs and their treatments are included in reference S1.

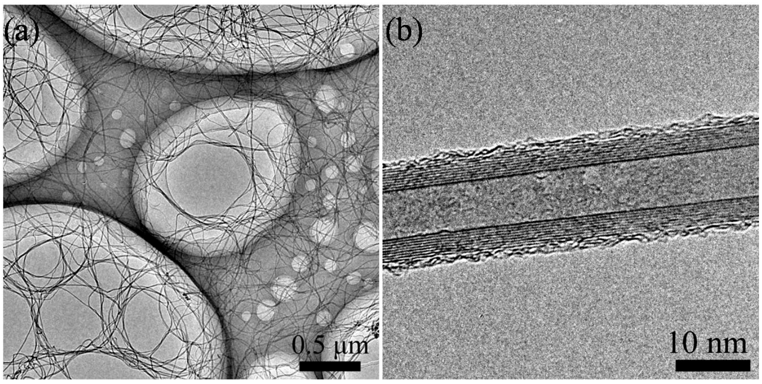

Figure S2 (a) Low- and (b) high- magnification TEM images of short-capillary CNTs. This kind of CNTs has the length up to 100 micrometers and diameter of ~11 nm. More details of such CNTs and their treatments are included in reference S2.

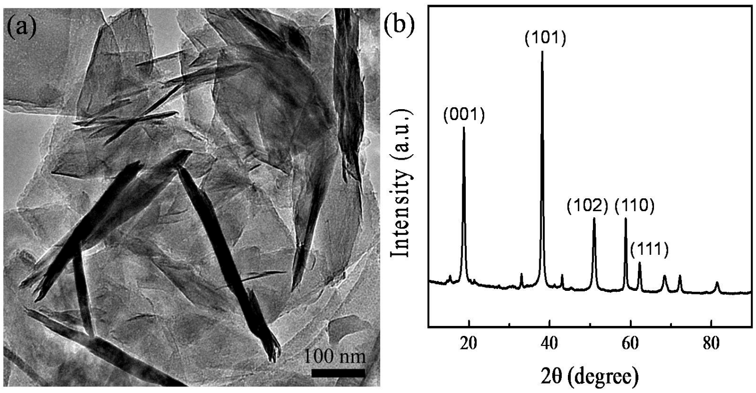

Figure S3 (a) TEM image and (b) XRD pattern of $Mg(OH)_2$ powder. It displays nanosheet morphology, with typical diffraction peaks.

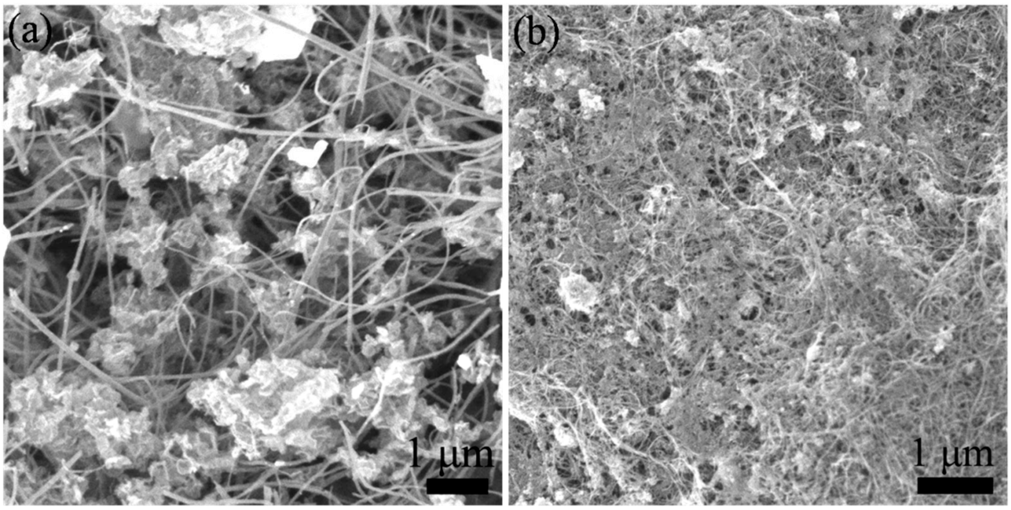

Figure S4 (a) SEM image of long-skeletal CNT/graphene nanocomposite, showing a loose phase separation. (b) SEM image of short-capillary CNT/graphene nanocomposite, showing tight integration with partial graphene aggregates.

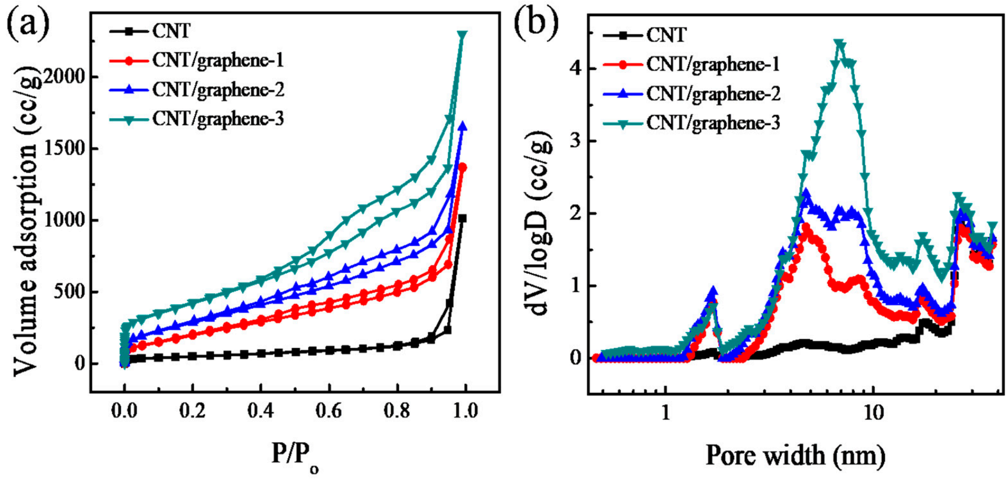

Figure S5 (a) N_2 sorption isotherms and (b) pore size distribution of the CNT and CNT/graphene composites with different compositions.

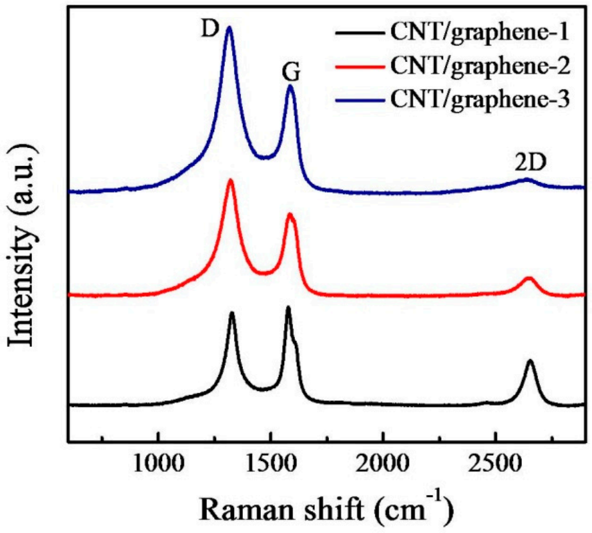

Figure S6 Raman spectrum of the as-prepared CNT/graphene composites.

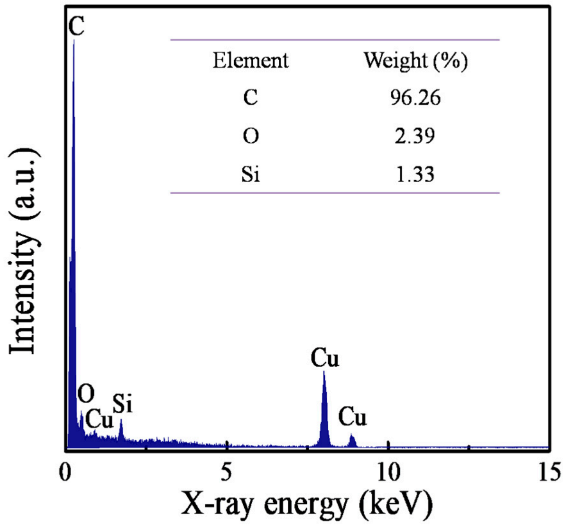

Figure S7. EDX spectrum of the skeletal-capillary CNT/graphene nanocomposite, showing the C and O lines, and Cu sample holder line.

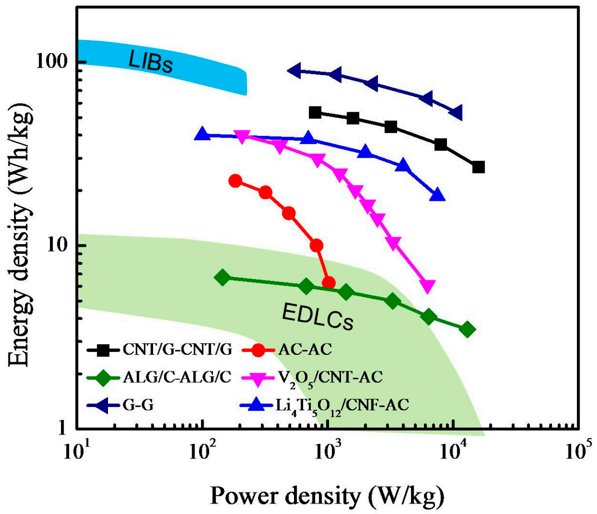

Figure S8 Comparison of Ragone plots of the ternary CNT/graphene supercapacitor with other devices, including symmetric supercapacitors made from ALG-C [S3], AC [S4] or graphene [S5], and asymmetric supercapacitors based on a V_2O_5/CNT [S4]or $Li_4Ti_5O_{12}$/CNF [S6] nanocomposite anode and AC cathode. All the data are based on the total mass of electrode materials.

Table S1 The composition and N_2 sorption characterization of the nanocomposites.

Sample	Composition (CNT skeleton:CNT capillary:$Mg(OH)_2$)	Composition* (CNT skeleton:CNT capillary:graphene, %)	Surface area (m^2/g)	Pore volume (cm^3/g)
CNTs	1:2:0	33:67:0	169.1	0.76
CNT/graphene-1	1:2:26	18:37:45	668.1	1.44
CNT/graphene-2	1:2:54	12:25:63	959.3	1.86
CNT/graphene-3	1:2:93	8:17:75	1453.7	2.72

* 9.5 wt-% carbon yield based on the weight of $Mg(OH)_2$.

References

[S1] Jia, X.; Chen, Z.; Suwarnasarn, A.; Rice, L.; Wang, X.; Sohn, H., Zhang, Q.; Wu, B.M.; Wei, F.; Lu, Y. High-performance flexible lithium-ion electrodes based on robust network architecture. *Energy Environ. Sci.* 2012, 5, 6845-6849.

[S2] Jia, X.; Zhang, Q.; Zhao, M.-Q.; Xu, G.-H.; Huang, J.-Q.; Qian, W., Lu, Y.; Wei, F. Dramatic enhancements in toughness of polyimide nanocomposite via long-CNT-induced long-range creep. *J. Mater. Chem.* 2012, 22, 7050-7056.

[S3] He, X.; Ling, P.; Qiu, J.; Yu, M.; Zhang, X.; Yu, C., Zheng, M. Efficient preparation of biomass-based mesoporous carbons for supercapacitors with both high energy density and high power density. *J. Power Sources* 2013, 240, 109-113.

[S4] Chen, Z.; Augustyn, V.; Wen, J.; Zhang, Y.; Shen, M.; Dunn, B., Lu, Y. High-Performance Supercapacitors Based on Intertwined CNT/V_2O_5 Nanowire Nanocomposites. *Adv Mater* 2011, 23, 791-795.

[S5] Kim, T.; Jung, G.; Yoo, S.; Suh, K.S.; Ruoff, R.S. Activated graphene-based carbons as supercapacitor electrodes with macro- and mesopores. *ACS Nano* 2013, 7, 6899-6905.

[S6] Naoi, K. 'Nanohybrid Capacitor': The Next Generation Electrochemical Capacitors. *Fuel Cells* 2010, 10, 825-833.
